# Emergence of ST463 *exoU*-Positive, Imipenem-Nonsusceptible Pseudomonas aeruginosa Isolates in China

**DOI:** 10.1128/spectrum.00105-23

**Published:** 2023-06-14

**Authors:** Ying Zhu, Yue Kang, Hui Zhang, Wei Yu, Ge Zhang, Jingjia Zhang, Wei Kang, Simeng Duan, Yingchun Xu, Qiwen Yang

**Affiliations:** a Clinical Laboratory Department, State Key Laboratory of Complex, Severe, and Rare Diseases, Peking Union Medical College Hospital, Chinese Academy of Medical Sciences, Beijing, China; b Graduate School, Peking Union Medical College, Chinese Academy of Medical Sciences, Beijing, China; c MRL Global Medical Affairs, MSD China, Shanghai, China; JMI Laboratories

**Keywords:** *Pseudomonas aeruginosa*, imipenem nonsusceptible, resistance mechanisms, *exoU*, ST463

## Abstract

This study investigated the resistance mechanisms and the distribution and proportions of virulence genes, including *exoU*, in 182 imipenem-nonsusceptible Pseudomonas aeruginosa (INS-PA) strains collected from China in 2019. There was no obvious prevalent sequence type or concentrated evolutionary multilocus sequence typing (MLST) type on the INS-PA phylogenetic tree in China. All of the INS-PA isolates harbored β-lactamases with/without other antimicrobial mechanisms, such as gross disruption of *oprD* and overexpression of efflux genes. Compared with *exoU*-negative isolates, *exoU*-positive isolates (25.3%, 46/182) presented higher virulence in A549 cell cytotoxicity assays. The southeast region of China had the highest proportion (52.2%, 24/46) of *exoU*-positive strains. The most frequent *exoU*-positive strains belonged to sequence type 463 (ST463) (23.9%, 11/46) and presented multiple resistance mechanisms and higher virulence in the Galleria mellonella infection model. The complex resistance mechanisms in INS-PA and the emergence of ST463 *exoU*-positive, multidrug-resistant P. aeruginosa strains in southeast China indicated a challenge that might lead to clinical treatment failure and higher mortality.

**IMPORTANCE** This study investigates the resistance mechanisms and distribution and proportions of virulence genes of imipenem-nonsusceptible Pseudomonas aeruginosa (INS-PA) isolates in China in 2019. Harboring PDC and OXA-50-like genes is discovered as the most prevalent resistance mechanism in INS-PA, and the virulence of *exoU*-positive INS-PA isolates was significantly higher than that of *exoU*-negative INS-PA isolates. There was an emergence of ST463 *exoU*-positive INS-PA isolates in Zhejiang, China, most of which presented multidrug resistance and hypervirulence.

## INTRODUCTION

Pseudomonas aeruginosa is one of the most common nosocomial pathogens worldwide. Carbapenem-resistant P. aeruginosa (CRPA) has been listed as a critical priority pathogen by the World Health Organization (WHO), and new antibiotics are urgently required to treat infected patients due to high global mortality (1). In 2020, China Antimicrobial Surveillance Network (CHINET) surveillance data revealed that the percentages of imipenem (IPM)-resistant and meropenem (MEM)-resistant P. aeruginosa isolates were 23.2% and 19.3%, respectively (2). The acquisition of carbapenem-hydrolyzing β-lactamases (such as Klebsiella pneumoniae carbapenemases [KPC], AIM, DIM, GIM, IMP, NDM, SPM, VIM, and OXA), overexpression of chromosome-encoded AmpC β-lactamase, acquisition of extended-spectrum AmpC cephalosporinases (ESACs), reduction of permeability of the outer membrane protein OprD, and overexpression of the major resistance-nodulation-division (RND) efflux pump systems (MexAB-OprM, MexCD-OprJ, MexEF-OprN, and MexXY-OprM) are all involved in carbapenem resistance in P. aeruginosa (3). The most common mechanism of imipenem resistance in P. aeruginosa is a combination of chromosomal AmpC production and a porin change. Indeed, a low level of AmpC enzyme production does not result in high-level carbapenem resistance, due to their limited potential to hydrolyze carbapenem drugs. However, their overproduction, together with reduced outer membrane porin permeability and/or efflux pump overexpression, contributes to high-level carbapenem resistance in this pathogen. P. aeruginosa can also obtain other β-lactamases by horizontal genetic transfer, including extended-spectrum β-lactamases (ESBLs), KPC, VIM, and metallo-β-lactamases (MBLs). The combination of these enzymes leads to high rates of carbapenem resistance in P. aeruginosa isolates (4).

Moreover, many Gram-negative bacteria, including P. aeruginosa, possess type III secretion systems (T3SS), which they utilize to introduce virulence factors directly into host cells. In P. aeruginosa, T3SS transport four secreted factors, *exoU*, *exoS*, *exoY*, and *exoT*. However, all of these factors may not be common to all P. aeruginosa strains. The *exoU* gene encodes a cytotoxic protein that rapidly destroys the cell membranes of mammalian cells by using its phospholipase activity (5). These virulence factors play important roles that may be involved in the genesis of acute lung injury, bacteremia, sepsis, and invasion of tissues. P. aeruginosa possesses a T3SS virulence mechanism (6).

Both carbapenem resistance and hypervirulence would probably result in poor clinical outcomes for infected patients. Therefore, this research is aimed at investigating the resistance mechanisms and distribution and proportions of virulence genes using whole-genome-sequencing techniques.

## RESULTS

### General information on P. aeruginosa isolates in 2019.

Of the isolates collected in 2019, 35 strains were proved to be contaminated in the process of whole-genome sequencing (WGS) and were not included in this analysis. The final 522 P. aeruginosa isolates that were taken into consideration were collected from intraabdominal tract infections (IAIs) (57/522), urinary tract infections (UTIs) (45/522), respiratory tract infections (RTIs) (374/522), and bloodstream infections (BSIs) (46/522) in 6 regions of China: central (45/522), east (104/522), northeast (63/522), south (77/522), southeast (153/522), and southwest (80/522). There were 340 isolates (patient age, 60.4 ± 18.7 years [mean ± standard deviation]) that were susceptible to imipenem and 182 isolates (patient age, 64.4 ± 16.8 years) that were nonsusceptible to imipenem. For imipenem-susceptible P. aeruginosa (IS-PA) isolates, 237 isolates were collected from males and 103 from females. For imipenem-nonsusceptible P. aeruginosa (INS-PA), 139 isolates were collected from males and 43 from females.

**FIG 1:**
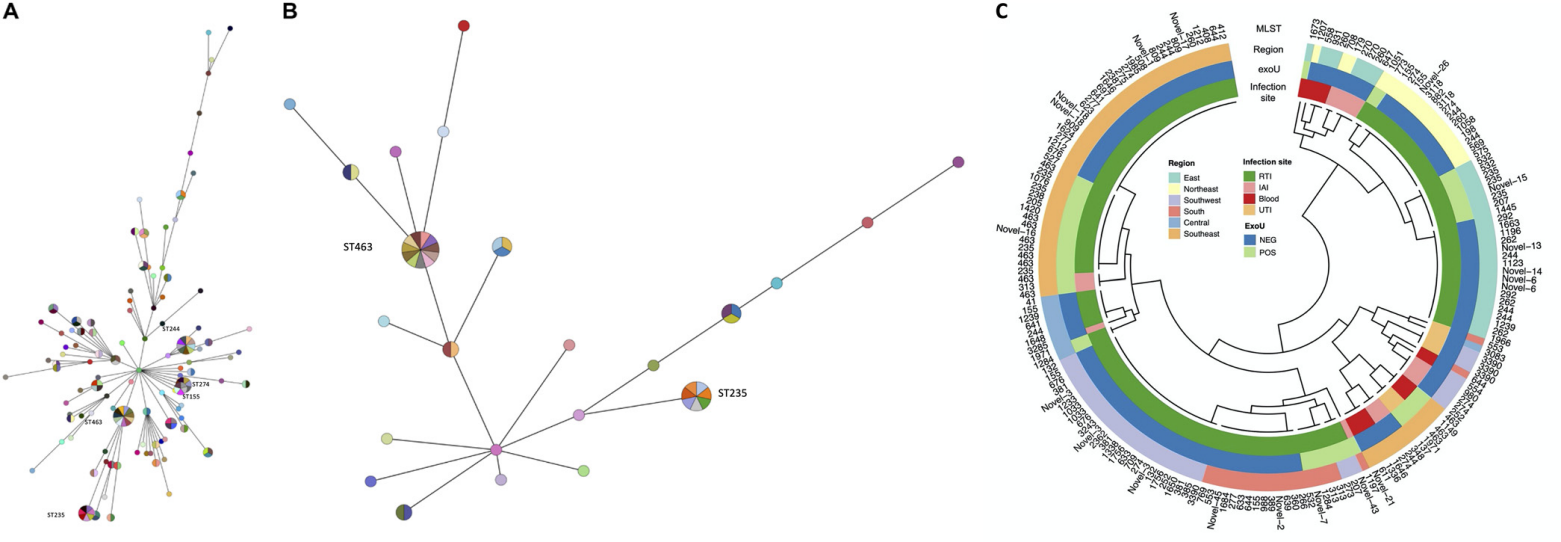
Phylogenetic trees of STs of all of the strains (A) and of *exoU*-positive strains (B) and of STs, virulence genes, different regions, and infection sites (C). (A and B) Minimum spanning trees of STs as determined by MLST, colored by strains. The size of each node reflects the number of isolates contained within the same clade. Each color represents one isolate. (C) MLST types of all of the 182 INS-PA isolates are listed outside the circle. MLST types, regions, *exoU*-positive or -negative status, and infection sites are marked with different colors from outside to inside. MLST, multilocus sequence typing; IAI, intra-abdominal tract infection; RTI, respiratory tract infection; UTI, urinary tract infection; NEG, negative; POS, positive.

According to the WGS results, there were 107 multilocus sequence typing (MLST) types detected among the 182 INS-PA isolates, with sequence type 463 (ST463) being the most frequently isolated (11/182), followed by ST244 (9/182), ST235 (7/182), and ST274 (7/182). The isolates did not present a concentrated evolutionary MLST type on the phylogenetic tree (Fig. 1A). There was no obvious prevalent sequence type in China in INS-PA isolates currently. Detailed information (STs, virulence genes, different regions, and infections) on all INS-PA isolates is shown in Fig. 1C.

### Antimicrobial susceptibility of P. aeruginosa isolates.

The antimicrobial susceptibilities of tested strains to common antibacterial agents are shown in Table 1. For IS-PA isolates, all of the antibacterial agents tested showed >70% susceptibility, except for piperacillin-tazobactam (TZP) and aztreonam (ATM). The rate of susceptibility of IS-PA isolates to aztreonam was 69.1%. Lower rates of susceptibility (<70%) were found for INS-PA isolates for all of the antibacterial agents tested, except for ceftolozane-tazobactam (C/T), amikacin (AMK), and tobramycin (TOB). However, INS-PA isolates from RTIs had higher rates of resistance to various antibacterial agents than INS-PA isolates from other infection sites on a numerical basis.

**TABLE 1:** Susceptibility rates and MIC distributions of common antibacterial agents against INS-PA and IS-PA isolates by infection site

Group of isolates, drug^*a*^	Value for^*b*^:														
	All indicated isolates			Indicated isolates from:											
				BSI			IAI			RTI			UTI		
	S (%)	MIC_50_	MIC_90_	S (%)	MIC_50_	MIC_90_	S (%)	MIC_50_	MIC_90_	S (%)	MIC_50_	MIC_90_	S (%)	MIC_50_	MIC_90_
INS-PA	*n* = 182			*n* = 14			*n* = 18			*n* = 142			*n* = 8		
Ceftazidime	46.7	16	>32	50	8	>32	56.3	8	>32	44.4	16	>32	50	8	>32
Cefepime	51.6	8	>32	57.1	8	>32	56.3	8	>32	50	8	>32	50	8	>32
Piperacillin-Tazobactam	24.7	>64	>64	21.4	>64	>64	18.8	>64	>64	23.9	>64	>64	37.5	>64	>64
Ceftolozane-Tazobactam	75.3	1	>32	78.6	1	>32	87.5	1	>32	74.6	1	>32	50	1	>32
Meropenem	20.3	8	>32	28.57	8	>32	25	8	16	18.31	8	>32	12.5	4	>32
Aztreonam	30.2	>16	>16	21.4	>16	>16	31.3	16	>16	31	>16	>16	12.5	>16	>16
Levofloxacin	31.3	2	>4	21.4	2	>4	43.8	2	>4	31	4	>4	12.5	>4	>4
Amikacin	86.3	<4	>32	100	<4	8	93.8	<4	16	85.2	<4	>32	62.5	8	>32
Tobramycin	84.1	<0.5	>8	100	<0.5	1	87.5	<0.5	>8	83.1	1	>8	62.5	1	>8
Colistin	81.3	2	4	64.3	2	4	93.8	2	2	81.7	2	4	87.5	2	4
IS-PA	*n* = 340			*n* = 32			*n* = 39			*n* = 232			*n* = 37		
Ceftazidime	75.6	4	>32	90.6	4	8	76.9	4	32	72.4	4	>32	81.1	4	32
Cefepime	84.1	4	16	96.9	2	8	87.2	4	16	80.6	4	16	91.9	4	8
Piperacillin-Tazobactam	58.8	8	>64	81.3	8	>64	64.1	8	>64	53.4	8	>64	67.6	8	>64
Ceftolozane-Tazobactam	94.7	1	4	100	0.5	1	94.9	1	2	93.5	1	4	97.3	1	1
Meropenem	93.8	0.5	2	0.5	1	<0.25	0.5	4	<0.25	0.5	2	<0.25	0.5	2	<0.25
Aztreonam	69.1	8	>16	90.6	8	8	69.2	8	>16	63.8	8	>16	83.8	8	16
Levofloxacin	77.9	<0.5	4	90.6	<0.5	1	89.7	<0.5	2	73.7	<0.5	4	81.1	<0.5	>4
Amikacin	99.4	<4	8	100	<4	<4	100	<4	<4	99.1	<4	8	100	<4	8
Tobramycin	98.2	<0.5	1	100	<0.5	1	100	<0.5	1	97.8	<0.5	1	97.3	<0.5	1
Colistin	82.4	2	4	81.3	2	4	89.7	2	4	81.5	2	4	81.1	2	4

a INS-PA, imipenem-nonsusceptible Pseudomonas aeruginosa; IS-PA, imipenem-susceptible Pseudomonas aeruginosa.

b S, susceptible; BSI, bloodstream infection; IAI, intraabdominal tract infection; RTI, respiratory tract infection; UTI, urinary tract infection.

### Resistance mechanism proportions and antimicrobial resistance gene distributions of INS-PA isolates by region.

**FIG 2:**
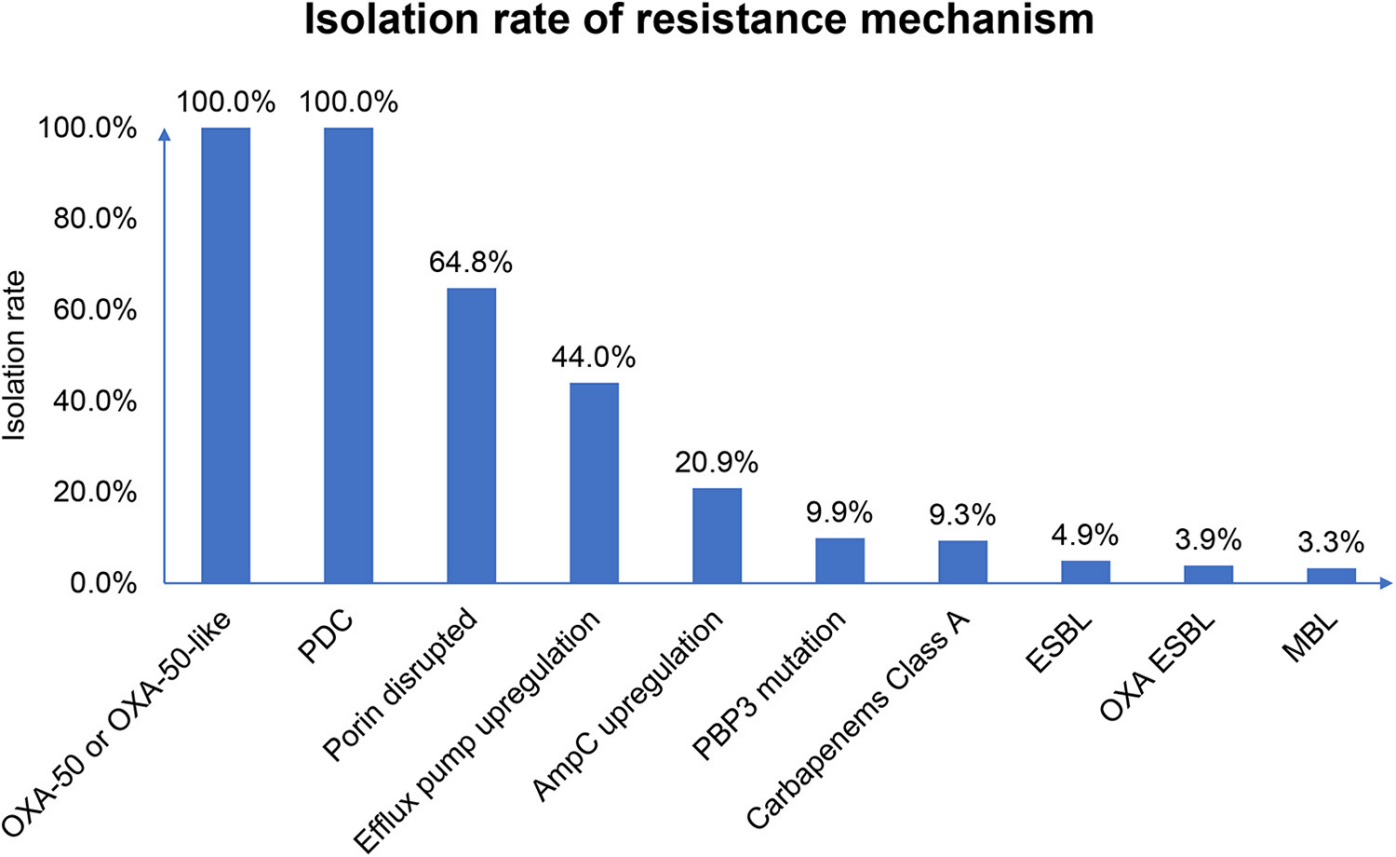
Proportion of each type of resistance mechanism. WGS was conducted to detect AMR and resistance mechanisms, while rates of upregulation were determined from publications in the literature that have demonstrated the loss or mutation of certain regulatory genes that cause upregulation. ESBL, extended-spectrum β-lactamase; OXA ESBL, OXA-10 or OXA-10-like; MBL, metallo β-lactamase.

**FIG 3:**
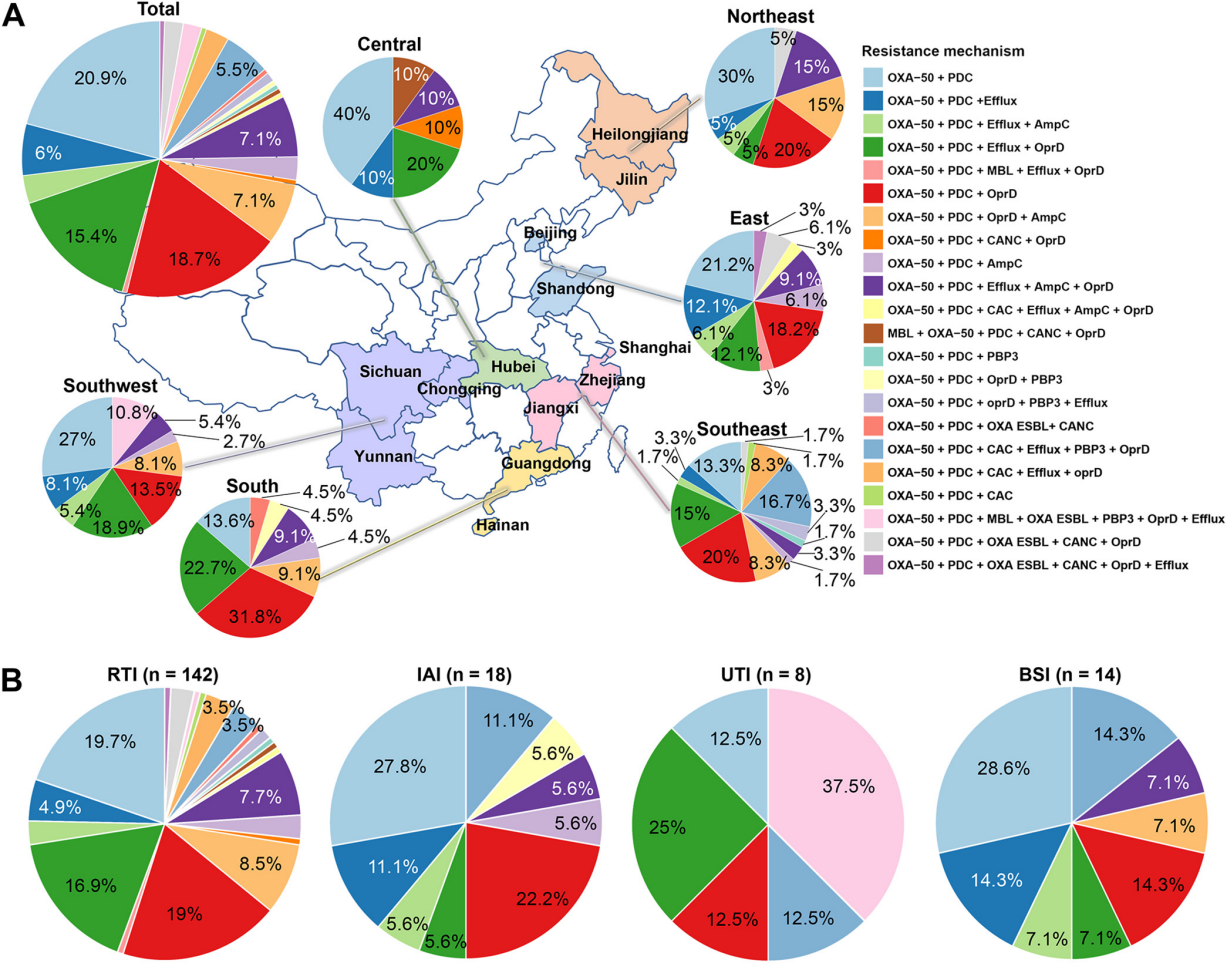
Distribution of resistance mechanisms by region (A) and infection site (B). Each color represents a combination of different kinds of resistance mechanisms. MBL, metallo β-lactamase; CAC, class A carbapenemase; OXA ESBL, OXA-10 or OXA-10-like; CANC, class A noncarbapenemase; RTI, respiratory tract infection; IAI, intraabdominal tract infection; UTI, urinary tract infection; BSI, bloodstream infection.

Antimicrobial resistance (AMR) gene analysis showed that all of the INS-PA isolates harbored *bla*_PDC_ and *bla*_OXA-50_ or *bla*_OXA-50-like_ (Fig. 2). In addition, 79.1% (144/182) of INS-PA strains harbored other AMR genes, such as *bla*_KPC-2_, *oprD* with a gross disruption (*oprD* gross disruption), or *mexR* or *nalD* loss/truncation, which indicated efflux upregulation combined with AMR genes *bla*_PDC_ and *bla*_OXA-50_ or *bla*_OXA-50-like_. Except for the generally encoded combination of *bla*_PDC_ and *bla*_OXA-50_ or *bla*_OXA-50-like_ (PDC+OXA-50/OXA-50-like) (38/182, 20.9%), the most common resistance mechanism combination was *bla*_PDC_, *bla*_OXA-50-like_, and *oprD* gross disruption (PDC+OXA-50-like+*oprD* gross disruption) (34/182, 18.7%), followed by PDC+OXA-50-like+*oprD* gross disruption+efflux upregulation (28/182, 15.4%). The distributions and proportions, however, were different in each region (Fig. 3). For the southeast region, 16.7% (10/60) of isolates had AMR genes comprising class A carbapenemase or ESBL genes (*bla*_KPC-2_, *bla*_GES-1_, or *bla*_GES-5_), *bla*_PDC_, *bla*_OXA-50_, or *bla*_OXA-50-like_, penicillin-binding protein 3 gene with mutation (PBP3 gene mutation), efflux upregulation genes, and *oprD* gross disruption simultaneously.

### Virulence gene analysis.

Among 182 INS-PA isolates, there were 46 strains that were *exoU* positive (25.3%). The distributions of *exoU*-positive isolates exhibited significant differences between regions (Table 2). The southeast region had the highest proportion and isolation rate of *exoU*-positive strains, which was significantly different from the other regions (*P = *0.001). The proportion of *exoU*-positive isolates with OXA-50-like+PDC+class A carbapenemase+efflux regulation+*oprD* gross disruption with or without the PBP3 gene mutation was significantly higher than that of *exoU*-negative isolates (*P ≤ *0.001) (Table 3).

**TABLE 2:** Comparison of characteristics between *exoU*-positive and *exoU*-negative isolates

Characteristic^*a*^	No. (%) of isolates that were:		*P* value
	*exoU* negative	*exoU* positive	
	*n* = 136	*n* = 46	
Patient age (yrs)			
<18	2 (1.47)	1 (2.17)	
≥18	134 (98.53)	45 (97.83)	1.000
Patient gender			
Female	33 (24.26)	10 (21.7)	
Male	103 (75.74)	36 (78.3)	0.730
Source			
BSI	10 (7.35)	4 (8.70)	1.000
IAI	12 (8.82)	4 (8.70)	1.000
RTI	107 (78.68)	35 (76.09)	0.714
UTI	5 (3.68)	3 (6.52)	0.691
Region			
Central	8 (5.88)	2 (4.35)	0.984
East	24 (17.65)	9 (19.57)	0.770
Northeast	18 (13.24)	2 (4.35)	0.096
South	16 (11.76)	6 (13.04)	0.818
Southeast	36 (26.47)	24 (52.17)	0.001
Southwest	34 (25)	3 (6.52)	0.007
Department			
Emergency room	7 (5.15)	2 (4.35)	1.000
ICU, general, unspecified	12 (8.82)	4 (8.70)	1.000
Medicine, general	56 (41.18)	14 (30.43)	0.195
Medicine, ICU	13 (9.56)	9 (19.57)	0.072
None given	2 (1.47)	0 (0)	0.993
Pediatric, general	1 (0.74)	0 (0)	1.000
Surgery, general	34 (25.00)	13 (28.26)	0.662
Surgery, ICU	11 (8.09)	4 (8.70)	1.000

a BSI, bloodstream infection; IAI, intraabdominal tract infection; RTI, respiratory tract infection; UTI, urinary tract infection; ICU, intensive care unit.

**TABLE 3:** Comparison of resistance mechanisms between *exoU*-positive and *exoU*-negative INS-PA isolates^*a*^

Resistance mechanisms	Value for isolates that were (*n* = 182):				*P* value
	*exoU* positive (*n* = 46)		*exoU* negative (*n* = 136)		
	No.	%	No.	%	
OXA-50 + PDC	4	8.7	34	25.0	0.019
OXA-50 + PDC + efflux	3	6.5	8	5.9	0.875
OXA-50 + PDC + efflux + *ampC*	0	0.0	6	4.4	0.332
OXA-50 + PDC + efflux + oprD	6	13.0	22	16.2	0.661
OXA-50 + PDC + MBL + efflux + *oprD*	1	2.2	0	0.0	0.568
OXA-50 + PDC + *oprD*	7	15.2	27	19.9	0.486
OXA-50 + PDC + *oprD* + *ampC*	0	0.0	13	9.6	0.065
OXA-50 + PDC + ESBL + OprD	0	0.0	1	0.7	1.000
OXA-50 + PDC + *ampC*	0	0.0	5	3.7	0.425
OXA-50 + PDC + efflux + *ampC* + *oprD*	3	6.5	10	7.4	0.850
OXA-50 + PDC + class A carbapenemase + efflux + *ampC* + *oprD*	1	2.2	0	0.0	0.568
OXA-50 + PDC + MBL + ESBL + *oprD*	0	0.0	1	0.7	1.000
OXA-50 + PDC + PBP3	0	0.0	1	0.7	1.000
OXA-50 + PDC + *oprD* + PBP3	1	2.2	0	0.0	0.568
OXA-50 + PDC + *oprD* + PBP3 + efflux	2	4.3	0	0.0	0.104
OXA-50 + PDC + OXA ESBL + ESBL	1	2.2	0	0.0	0.568
OXA-50 + PDC + class A carbapenemase + efflux + PBP3 + *oprD*	10	21.7	0	0.0	<0.001
OXA-50 + PDC + class A carbapenemase + efflux + *oprD*	5	10.9	0	0.0	0.001
OXA-50 + PDC + class A carbapenemase	0	0.0	1	0.7	1.000
OXA-50 + PDC + MBL + OXA ESBL + PBP3 + OprD + efflux	0	0.0	4	2.9	0.552
OXA-50 + PDC + OXA ESBL + ESBL + *oprD*	2	4.3	2	1.5	0.569
OXA-50 + PDC + OXA ESBL + ESBL + *oprD* + efflux	0	0.0	1	0.7	1.000

a INS-PA, imipenem-nonsusceptible Pseudomonas aeruginosa.

### Virulence of *exoU*-positive strains.

**FIG 4:**
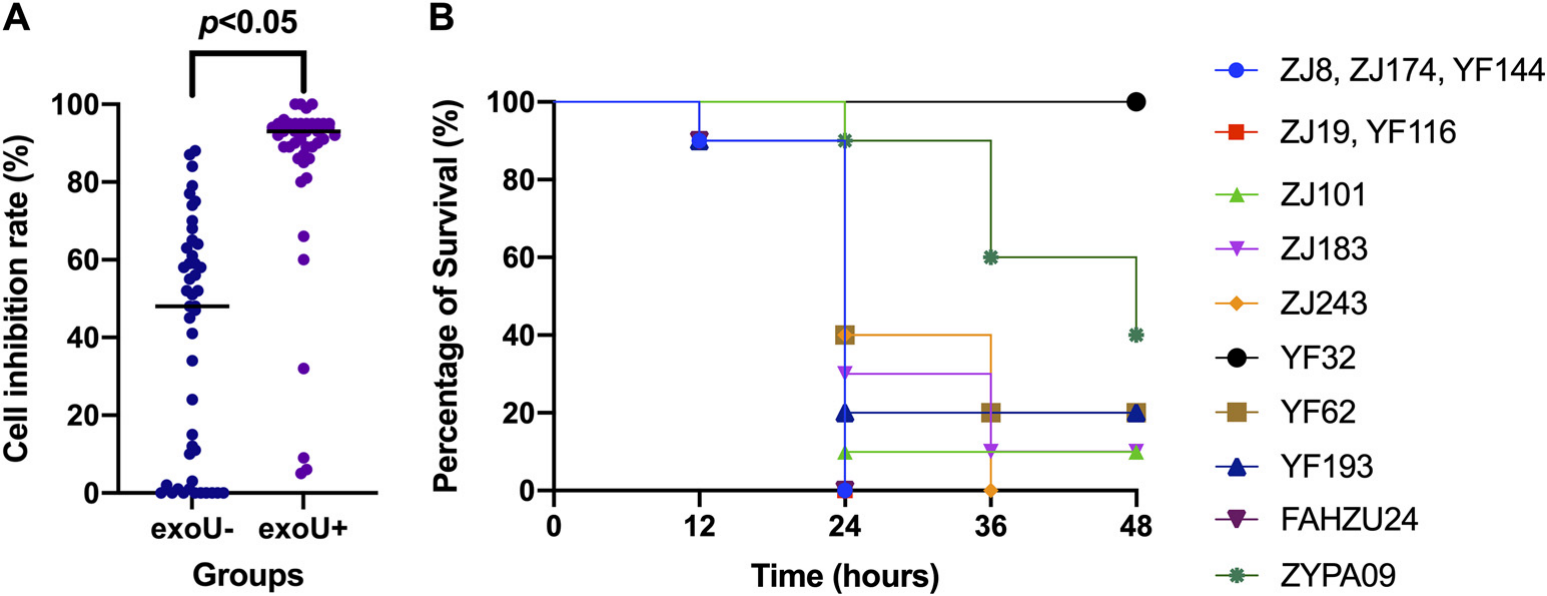
Virulence tests of ST463 strains with or without *exoU*. (A) Cell inhibition rates of 46 *exoU*-positive and 46 *exoU*-negative strains in A549 human pulmonary adenocarcinoma cell cytotoxicity assays. (B) Probability of survival of larvae infected with 11 ST463 *exoU*-positive strains from Zhejiang in the Galleria mellonella larva infection model. Strain FAHZU24 was the hypervirulent control, and strain ZYPA09 the hypovirulent control.

The cytotoxicity assay (Fig. 4) showed that the cell inhibition rates of *exoU*-negative strains were significantly lower than those of *exoU*-positive strains (*P* < 0.05). Most cell inhibition rates of *exoU*-positive strains were >92% (the inhibition rate of the hypervirulent control strain, FAHZU24), and some even reached 100%. The cell inhibition rates of *exoU*-negative strains were mostly <78% (the inhibition rate of the hypovirulent control strain, ZYPA09), with some even reduced to 0%.

### Virulence and resistance analysis of ST463 strains.

All of the ST463 strains (*n* = 11) collected from Zhejiang province were *exoU* positive. Six of the 11 ST463 strains were collected from intensive care units (ICUs), and the patient ages ranged from 42 to 72 years (Table 4). Two of the 11 ST463 strains were collected from blood samples, which was significantly higher than for non-ST463 strains (12/171, *P < *0.05). All of these ST463 isolates harbored *bla*_OXA-486_, *bla*_PDC-8_ combined with PBP3 gene mutation, *oprD* gross disruption, and efflux pump upregulation, and 10 of them harbored *bla*_KPC-2_ (Table 4). The imipenem MICs of the 11 strains were all >32 μg/mL, presenting high-level resistance to imipenem.

**TABLE 4:** Characteristics of the 11 ST463 strains

Strain	Patient		Source^*a*^	Department	Beta-lactamases	PBP3 mutation	AmpC upregulation	*oprD*	Efflux pump upregulation
	Age	Gender							
1	66	Male	RTI	Surgery, ICU	OXA-486, PDC-8	F533L^*b*^	No	Gross disruption	Yes (*mexZ*, *mexXY*)
2	48	Female	IAI	Surgery, general	KPC-2, OXA-486, PDC-8	F533L	No	Gross disruption	Yes (*mexZ*, *mexXY*)
3	74	Male	RTI	Medicine, ICU	KPC-2, OXA-486, PDC-8	F533L	No	Gross disruption	Yes (*mexZ*, *mexXY*)
4	44	Male	BSI	Medicine, general	KPC-2, OXA-486, PDC-8	F533L	No	Gross disruption	Yes (*mexZ*, *mexXY*)
5	72	Female	UTI	Medicine, ICU	KPC-2, OXA-486, PDC-8	F533L	No	Gross disruption	Yes (*mexZ*, *mexXY*)
6	59	Male	RTI	Medicine, ICU	KPC-2, OXA-486, PDC-8	F533L	No	Gross disruption	Yes (*mexZ*, *mexXY*)
7	54	Male	RTI	Surgery, ICU	KPC-2, OXA-486, PDC-8	F533L	No	Gross disruption	Yes, (*mexZ*, *mexXY*)
8	72	Female	RTI	Emergency room	KPC-2, OXA-486, PDC-8	F533L	No	Gross disruption	Yes, (*mexZ*, *mexXY*)
9	56	Male	RTI	Medicine, ICU	KPC-2, OXA-486, PDC-8	F533L	No	Gross disruption	Yes, (*mexZ*, *mexXY*)
10	42	Female	IAI	Surgery, general	KPC-2, OXA-486, PDC-8	F533L	No	Gross disruption	Yes, (*mexZ*, *mexXY*)
11	69	Female	BSI	Medicine, general	KPC-2, OXA-486, PDC-8	F533L	No	Gross disruption	Yes, (*mexZ*, *mexXY*)

a RTI, respiratory tract infection; IAI, intraabdominal tract infection; BSI, bloodstream infection; UTI, urinary tract infection.

b F533L, a change of F to L at position 533.

In the Galleria mellonella larva infection model, the virulence of 63.6% (7/11) ST463 *exoU*-positive strains showed no significant difference (*P* < 0.05) from that of the hypervirulent control strain, FAHZU24 (Fig. 4), and that of 36.4% (4/11) showed significant differences (*P* < 0.05) from the virulence of both FAHZU24 and the hypovirulent control, ZYPA09. In all, 63.6% (7/11) of ST463 *exoU*-positive strains were as virulent as the hypervirulent control, FAHZU24, and 27.3% (3/11) showed medium virulence (between that of the hypervirulent control, FAHZU24, and the hypovirulent control, ZYPA09). Only 9.1% (1/11) of the ST463, *exoU*-positive strains showed hypovirulence similar to that of the hypovirulent control, ZYPA09.

Most of the ST463 *exoU*-positive strains collected from Zhejiang presented carbapenem resistance and hypervirulence. However, the evolutionary relationship was not obviously concentrated, based on the phylogenetic tree (Fig. 1B).

## DISCUSSION

The resistance mechanisms of INS-PA are complex and associated with several AMR genes or resistance mechanisms. Because all of the isolates in this research were imipenem-nonsusceptible strains rather than meropenem- or ertapenem-nonsusceptible isolates, the proportion of porin loss was higher than that of efflux pump upregulation numerically. The rate of metallo-β-lactamase (MBL)-harboring isolates was low, and most of the MBL-harboring isolates were collected from the southwest (4/6).

The sequence type (ST) distribution varied from region to region. ST235 has been reported as the most prevalent sequence type in single-center research conducted in the southwest over a 10-year period (7). It is noteworthy that ST235 was not detected in the southwest in the present study but was found in 4 isolates from the southeast and 3 from the east. ST235 with the hypervirulence gene is the most prevalent sequence type, with clones categorized as high-risk and widespread associated with poor clinical outcomes. In part, this is due to multilevel and high-level antibiotic resistance (8). In the present research, the ST463 strains that were isolated from Zhejiang Province had the highest proportion. Other research from Zhejiang also reported that ST463 with coexistence of *exoS* and *exoU* was the most prevalent ST type (9) and presented both multidrug resistance (MDR) and hypervirulence (10). Ten of the 11 ST463 isolates in this research harbored *bla*_KPC-2_, resulting in high MICs for most of the β-lactam antibiotics tested, such as ceftazidime (CAZ) (>32 mg/L), cefepime (FEP) (>32 mg/L), piperacillin-tazobactam (>64 mg/L), ceftolozane-tazobactam (>32 mg/L), and meropenem (>32 mg/L, except for one isolate). As there was no obvious evolutionary relationship observed, the ST463 strains collected from the same place tend to be the prevalent type rather than outbreak strains. The clone of ST463 should be noted in clinical practice as high risk, because it contains both the virulence gene and AMR genes.

The present study found that there was no prevalent ST clone of INS-PA nationwide. Therefore, INS-PA is not spreading as a resistant clone in China. However, it should be pointed out that the higher proportions of *exoU*-positive isolates and ST463 strains in the southeast region could indicate that a resistant clone is spreading within certain regions. This finding was different from reported results from other countries, which had prevalent clones of resistant or high-risk P. aeruginosa, such as ST111, ST175, and ST235 (11). For example, ST175 is the most frequent extremely drug-resistant (XDR) high-risk clone detected in Spanish hospitals (12). However, the emergence of ST463 *exoU*-positive, multidrug-resistant P. aeruginosa strains in east China also indicates a challenge that may lead to failures of clinical treatment and a higher mortality rate. One retrospective cohort study in eastern China found that ST463 was predominant (48.0%) among 50 CRPA BSI cases and that the 28-day mortality was significantly higher for ST463 cases than for non-ST463 cases (66.7% versus 33.3%, *P = *0.03) (13, 14). The reason given for the presence of ST463 with poorer outcomes was explained in the publication (14). Infections related to *exoU*-producing strains have been found to be associated with more severe clinical symptoms and poorer outcomes than infections caused by *exoS*-positive (*exoU*-negative) isolates (6). Moreover, drug-resistant P. aeruginosa strains, especially CRPA, contributed to poorer clinical outcomes simultaneously. One meta-analysis demonstrated a >2-fold-increased risk of mortality with multidrug-resistant P. aeruginosa (MDR-PA) (relative risk [RR], 2.34; 95% confidence interval [CI], 1.53 to 3.57) and a prolonged length of hospitalization compared to the risk of mortality and length of hospitalization from infections with susceptible P. aeruginosa strains (15). Thus, *exoU* and *exoS* virulence genes coexisting with the *bla*_KPC_ resistance gene in ST463 CRPA may be an important intrinsic cause of the poor prognosis of clinical P. aeruginosa BSIs (14). Therefore, infections caused by hypervirulent and carbapenem-resistant organisms have clinical and economic consequences. Hospital-acquired resistant and MDR P. aeruginosa infections will probably result in poorer clinical outcomes, and it will be necessary to rigorously monitor patients in clinical practice.

The INS-PA strains were isolated from 13 provinces, which may not represent the whole of China, because both sequencing types and resistance mechanisms are different across regions. The IS-PAs were not tested by WGS, which may make the relationship between genotype and phenotype unreliable.

## MATERIALS AND METHODS

### Isolates from SMART in 2019.

All of the P. aeruginosa isolates were collected during the Study for Monitoring Antimicrobial Resistance Trends (SMART) (16), which isolated pathogens from abdominal, urinary tract, blood, and respiratory tract specimens of patients from 16 hospitals in six regions (central, east, northeast, south, southeast, and southwest) across China in 2019. The central clinical microbiology laboratory of the Peking Union Medical College Hospital reidentified all of the isolates using matrix-assisted laser desorption ionization–time of flight mass spectrometry (MALDI-TOF MS) (Vitek MS; bioMérieux, France). The Human Research Ethics Committee of Peking Union Medical College Hospital approved the study protocols (no. S-K238).

### Antimicrobial susceptibility testing.

Antimicrobial susceptibility testing was performed at the Peking Union Medical College Hospital central laboratory using panels purchased from Thermo Fisher Scientific (Cleveland, OH, USA). MICs were interpreted using the CLSI breakpoints (17) (all antimicrobial agents except colistin [COL]) or the EUCAST breakpoint (colistin) (18).

The antimicrobial agents tested included imipenem (IPM), meropenem (MEM), ceftazidime (CAZ), cefepime (FEP), piperacillin-tazobactam (TZP), ceftolozane-tazobactam (C/T), aztreonam (ATM), levofloxacin (LVX), amikacin (AMK), tobramycin (TOB), and colistin (COL). P. aeruginosa ATCC 27853 was used as the quality control (QC) strain for each batch of MIC tests.

### Cytotoxicity assay.

Cytotoxicity assays using A549 human pulmonary adenocarcinoma cells were conducted on all 46 *exoU*-positive strains and 46 *exoU*-negative strains selected according to region to evaluate the virulence. The high-cytotoxicity control was the *exoU*-positive/*exoS*-negative, ST235 Pseudomonas aeruginosa strain FAHZU24, and the low-cytotoxicity control strain was the *exoU*-negative/*exoS*-positive, ST236 strain ZYPA09. The cells were cultured in F-12K medium with 10% fetal bovine serum (FBS) at 37°C with 5% CO_2_. Amounts of 100 μL of fresh medium with 6 × 10^3^ cells/well were plated in 96-well plates and cultured for 24 h. Then, overnight cultures of single colonies from agar plates were diluted 10^3^ times (3 × 10^6^ CFU/mL) with F-12K medium (including 10% FBS). One hundred microliters diluted bacterial culture was added into each well (multiplicity of infection of 50) and cultured at 37°C with 5% CO_2_ for 3 h. Then, the supernatant was discarded and the cells washed with 100 μL fresh medium. Finally, 100 μL fresh F-12K medium (including 10% FBS) and 10 μL cell counting kit-8 (CCK-8) solution was added. The optical density at 450 nm (OD_450_) was detected after 2.5 h of culture. Each group had 6 compound wells. The inhibition rate was calculated as follows: (*A*_control_ − *A*_experiment_)/(*A*_control_ − *A*_blank_) × 100, where *A* is absorbance. A higher inhibition rate indicated stronger cytotoxicity. GraphPad Prism 9 software was employed for statistical analysis, and a *P* value of <0.05 was considered significant.

### Galleria mellonella larva infection model.

The virulence of *exoU*-positive ST463 strains was also tested by the Galleria mellonella larva infection model. Normal saline was used to adjust the bacterial suspension to 1 × 10^6^ CFU/mL, and 10 μL bacterial suspension was injected into each larva (*n* = 10 larvae/strain). Then, the larvae were incubated at 35°C and the number of surviving larvae was recorded once every 12 h for 48 h. Strain FAHZU24 is referred to as the hypervirulent control, and ZYPA09 as the hypovirulent control (10).

### WGS.

All imipenem-nonsusceptible isolates were sent for whole-genome sequencing (WGS). Bacteria cultured to stationary phase from single colonies were pelleted in 1× Tris-EDTA buffer. DNA isolation used magnetic bead chemistry, and library preparation used the in-house method of Beijing Genomics Institute (BGI, Wuhan, China). Libraries were sequenced on a high-throughput Illumina sequencer in a 2 × 150-bp paired-end configuration to a calculated coverage depth of ×100.

### WGS analysis.

The CLC Genomics Workbench (Qiagen) was used for WGS analysis. On acquisition of fastq files, reads were trimmed for quality and adapter sequences, sampled to approximately ×100 coverage depth, and assembled *de novo* for downstream analysis. Genes encoding β-lactamases were identified by screening assemblies using the ResFinder database (https://cge.food.dtu.dk/services/ResFinder/), downloaded on 9 July 2020. Coverage and identity cutoffs were set to ≥35% and ≥72%, respectively, though any positively identified antimicrobial resistance gene that was <100% for either parameter was confirmed by read mapping and/or examination at the amino acid level to identify a β-lactamase variant. Various genes of interest were analyzed for gross disruptions or previously characterized mutations by pairwise alignment to a reference sequence. *oprD* was tested for permeability, and the PBP3 gene (*ftsI*) was tested for target mutation. For detecting *ampC* regulation, *ampD*, *ampDh2*, *ampDh3*, *dacB* (*pbp4*), *mpl*, *nuoN*, and *ampR* were analyzed. To detect efflux regulation, *nalD*, *mexR*, *nalC*, and *mexZ* were analyzed. The *exoU* gene was tested for hypervirulence (19–29). Multilocus sequence typing determination was performed using CLC Genomics with schema downloaded from Pubmlst.org on 4 November 2020. The phylogenetic tree was constructed based on MLST of each isolate using GrapeTree (version 1.4.0, https://github.com/achtman-lab/GrapeTree) and clustered based on MLST, region, *exoU*-positive/-negative status, and infection site using the circlize package in R Studio (version 4.0.5).

### Statistical analysis.

SPSS (version 17.0; IBM) was used for all statistical analysis. Descriptive analysis was performed to calculate the susceptibility and proportion of each resistance mechanism. Student’s *t* test was performed for the cytotoxicity assay for comparison between the cell inhibition rates of the *exoU*-positive group and the *exoU*-negative group. The Gehan-Breslow-Wilcoxon test was performed on the survival curves of clinical strains and control strains. Comparison of the resistance mechanisms and clinical characteristics between the *exoU*-positive and *exoU*-negative groups was evaluated using the chi-square test. *P* values of <0.05 were considered to be statistically significant findings.

## References

[1] Mohamed A, Daef E, Nafie A, Shaban L, Ibrahim M. 2021. Characteristics of carbapenem-resistant gram-negative bacilli in patients with ventilator-associated pneumonia. Antibiotics (Basel) 10:1325. doi:10.3390/antibiotics10111325. 34827263 PMC8615042

[2] CHINET. 2020. China surveillance of bacteria resistance: results of 2020. China Antimicrobial Surveillance Network. http://www.chinets.com/Document. Accessed 30 December 2021.

[3] Kao C-Y, Chen S-S, Hung K-H, Wu H-M, Hsueh P-R, Yan J-J, Wu J-J. 2016. Overproduction of active efflux pump and variations of OprD dominate in imipenem-resistant Pseudomonas aeruginosa isolated from patients with bloodstream infections in Taiwan. BMC Microbiol 16:107. doi:10.1186/s12866-016-0719-2. 27296461 PMC4906909

[4] Santajit S, Indrawattana N. 2016. Mechanisms of antimicrobial resistance in ESKAPE pathogens. Biomed Res Int 2016:2475067. doi:10.1155/2016/2475067. 27274985 PMC4871955

[5] Subedi D, Vijay AK, Kohli GS, Rice SA, Willcox M. 2018. Association between possession of ExoU and antibiotic resistance in Pseudomonas aeruginosa. PLoS One 13:e0204936. doi:10.1371/journal.pone.0204936. 30265709 PMC6161911

[6] Elabbadi A, Pont S, Verdet C, Plésiat P, Cretin F, Voiriot G, Fartoukh M, Djibré M. 2020. An unusual community-acquired invasive and multi systemic infection due to ExoU-harboring Pseudomonas aeruginosa strain: clinical disease and microbiological characteristics. J Microbiol Immunol Infect 53:647–651. doi:10.1016/j.jmii.2019.06.008. 31345686

[7] Feng W, Huang Q, Wang Y, Yuan Q, Li X, Xia P, Sun F. 2021. Changes in the resistance and epidemiological characteristics of Pseudomonas aeruginosa during a ten-year period. J Microbiol Immunol Infect 54:261–266. doi:10.1016/j.jmii.2019.08.017. 31628088

[8] Treepong P, Kos VN, Guyeux C, Blanc DS, Bertrand X, Valot B, Hocquet D. 2018. Global emergence of the widespread Pseudomonas aeruginosa ST235 clone. Clin Microbiol Infect 24:258–266. doi:10.1016/j.cmi.2017.06.018. 28648860

[9] Hu Y, Peng W, Wu Y, Li H, Wang Q, Yi H, Zhang R, Shao B, Zhu K. 2021. A potential high-risk clone of Pseudomonas aeruginosa ST463. Front Microbiol 12:670202. doi:10.3389/fmicb.2021.670202. 34122384 PMC8193091

[10] Zhang P, Wang J, Li Y, Shi W, Cai H, Yang Q, Li X, Yu Y, Qu T, Jiang Y. 2022. Emergence of bla(KPC-33)-harboring hypervirulent ST463 Pseudomonas aeruginosa causing fatal infections in China. J Infect 85:e86–e88. doi:10.1016/j.jinf.2022.07.011. 35863519

[11] Pérez A, Gato E, Pérez-Llarena J, Fernández-Cuenca F, Gude MJ, Oviaño M, Pachón ME, Garnacho J, González V, Pascual Á, Cisneros JM, Bou G. 2019. High incidence of MDR and XDR Pseudomonas aeruginosa isolates obtained from patients with ventilator-associated pneumonia in Greece, Italy and Spain as part of the MagicBullet clinical trial. J Antimicrob Chemother 74:1244–1252. doi:10.1093/jac/dkz030. 30753505

[12] Del Barrio-Tofiño E, Zamorano L, Cortes-Lara S, López-Causapé C, Sánchez-Diener I, Cabot G, Bou G, Martínez-Martínez L, Oliver A, GEMARA-SEIMC/REIPI Pseudomonas Study Group. 2019. Spanish nationwide survey on Pseudomonas aeruginosa antimicrobial resistance mechanisms and epidemiology. J Antimicrob Chemother 74:1825–1835. doi:10.1093/jac/dkz147. 30989186

[13] David S, Reuter S, Harris SR, Glasner C, Feltwell T, Argimon S, Abudahab K, Goater R, Giani T, Errico G, Aspbury M, Sjunnebo S, Feil EJ, Rossolini GM, Aanensen DM, Grundmann H, ESGEM Study Group. 2019. Epidemic of carbapenem-resistant Klebsiella pneumoniae in Europe is driven by nosocomial spread. Nat Microbiol 4:1919–1929. doi:10.1038/s41564-019-0492-8. 31358985 PMC7244338

[14] Hu H, Zhang Y, Zhang P, Wang J, Yuan Q, Shi W, Zhang S, Feng H, Chen Y, Yu M, Chen H, Jiang Y, Yang Q, Qu T. 2021. Bloodstream infections caused by Klebsiella pneumoniae carbapenemase-producing P. aeruginosa sequence type 463, associated with high mortality rates in China: a retrospective cohort study. Front Cell Infect Microbiol 11:756782. doi:10.3389/fcimb.2021.756782. 34790589 PMC8592259

[15] Nathwani D, Raman G, Sulham K, Gavaghan M, Menon V. 2014. Clinical and economic consequences of hospital-acquired resistant and multidrug-resistant Pseudomonas aeruginosa infections: a systematic review and meta-analysis. Antimicrob Resist Infect Control 3:32. doi:10.1186/2047-2994-3-32. 25371812 PMC4219028

[16] Lob SH, Karlowsky JA, Young K, Motyl MR, Hawser S, Kothari ND, Sahm DF. 2020. In vitro activity of imipenem-relebactam against resistant phenotypes of Enterobacteriaceae and Pseudomonas aeruginosa isolated from intraabdominal and urinary tract infection samples—SMART Surveillance Europe 2015–2017. J Med Microbiol 69:207–217. doi:10.1099/jmm.0.001142. 31976856

[17] CLSI. 2023. M100. Performance standards for antimicrobial susceptibility testing, 33rd ed. CLSI, Wayne, PA.

[18] EUCAST. 2023. Breakpoint tables for interpretation of MICs and zone diameters, version 13.0. https://www.eucast.org/clinical_breakpoints.

[19] Quale J, Bratu S, Gupta J, Landman D. 2006. Interplay of efflux system, ampC, and oprD expression in carbapenem resistance of Pseudomonas aeruginosa clinical isolates. Antimicrob Agents Chemother 50:1633–1641. doi:10.1128/AAC.50.5.1633-1641.2006. 16641429 PMC1472219

[20] Torrens G, Hernández SB, Ayala JA, Moya B, Juan C, Cava F, Oliver A. 2019. Regulation of AmpC-driven beta-lactam resistance in Pseudomonas aeruginosa: different pathways, different signaling. mSystems 4:e00524-19. doi:10.1128/mSystems.00524-19. 31796566 PMC6890930

[21] Kos VN, McLaughlin RE, Gardner HA. 2016. Elucidation of mechanisms of ceftazidime resistance among clinical isolates of Pseudomonas aeruginosa by using genomic data. Antimicrob Agents Chemother 60:3856–3861. doi:10.1128/AAC.03113-15. 27067331 PMC4879354

[22] Tsutsumi Y, Tomita H, Tanimoto K. 2013. Identification of novel genes responsible for overexpression of ampC in Pseudomonas aeruginosa PAO1. Antimicrob Agents Chemother 57:5987–5993. doi:10.1128/AAC.01291-13. 24041903 PMC3837884

[23] Cabot G, Ocampo-Sosa AA, Domínguez MA, Gago JF, Juan C, Tubau F, Rodríguez C, Moyà B, Peña C, Martínez-Martínez L, Oliver A, Spanish Network for Research in Infectious Diseases (REIPI). 2012. Genetic markers of widespread extensively drug-resistant Pseudomonas aeruginosa high-risk clones. Antimicrob Agents Chemother 56:6349–6357. doi:10.1128/AAC.01388-12. 23045355 PMC3497190

[24] Caille O, Zincke D, Merighi M, Balasubramanian D, Kumari H, Kong K-F, Silva-Herzog E, Narasimhan G, Schneper L, Lory S, Mathee K. 2014. Structural and functional characterization of Pseudomonas aeruginosa global regulator AmpR. J Bacteriol 196:3890–3902. doi:10.1128/JB.01997-14. 25182487 PMC4248820

[25] Balasubramanian D, Kumari H, Mathee K. 2015. Pseudomonas aeruginosa AmpR: an acute-chronic switch regulator. Pathog Dis 73:1–14. doi:10.1111/2049-632X.12208. PMC4542883 25066236

[26] Morita Y, Cao L, Gould VC, Avison MB, Poole K. 2006. nalD encodes a second repressor of the mexAB-oprM multidrug efflux operon of Pseudomonas aeruginosa. J Bacteriol 188:8649–8654. doi:10.1128/JB.01342-06. 17028276 PMC1698243

[27] Hay T, Fraud S, Lau CHF, Gilmour C, Poole K. 2013. Antibiotic inducibility of the mexXY multidrug efflux operon of Pseudomonas aeruginosa: involvement of the MexZ anti-repressor ArmZ. PLoS One 8:e56858. doi:10.1371/journal.pone.0056858. 23441219 PMC3575510

[28] Han S, Zaniewski RP, Marr ES, Lacey BM, Tomaras AP, Evdokimov A, Miller JR, Shanmugasundaram V. 2010. Structural basis for effectiveness of siderophore-conjugated monocarbams against clinically relevant strains of Pseudomonas aeruginosa. Proc Natl Acad Sci USA 107:22002–22007. doi:10.1073/pnas.1013092107. 21135211 PMC3009787

[29] del Barrio-Tofiño E, López-Causapé C, Cabot G, Rivera A, Benito N, Segura C, Montero MM, Sorlí L, Tubau F, Gómez-Zorrilla S, Tormo N, Durá-Navarro R, Viedma E, Resino-Foz E, Fernández-Martínez M, González-Rico C, Alejo-Cancho I, Martínez JA, Labayru-Echverria C, Dueñas C, Ayestarán I, Zamorano L, Martinez-Martinez L, Horcajada JP, Oliver A. 2017. Genomics and susceptibility profiles of extensively drug-resistant Pseudomonas aeruginosa isolates from Spain. Antimicrob Agents Chemother 61:e01589-17. doi:10.1128/AAC.02352-17. 28874376 PMC5655108

